# News and Coming Events

**Published:** 1909-05-29

**Authors:** 


### News and Coming Events.

The Council for the Promotion of the Higher Training of Midwives held its annual meeting of subscribers and friends at The Deanery, St. Paul's, on Tuesday, May 25, at 3.15 p.m., when the chair was taken by Lady George Hamilton. The Venerable Archdeacon of Lewisham moved the adoption of the report of the Home for Mothers and Babies for the past year, and Miss Jane Walker, M.D., and Miss Alice Gregory each addressed the company.

---

The National Society for the Prevention of Cruelty to Children (incorporated by Royal Charter) will hold a great meeting in the Mansion House, London, on Monday, May 24, at 3.30 p.m., when the Right Hon. the Lord Mayor will preside, and the speakers will include the Earl of Ancaster, Lord Alverstone (Lord Chief Justice of England), the Right Hon. Herbert Samuel, M.P., Sir Francis Channing, Bart., M.P., the Bishop of Kensington, and the Rev. Father Bernard Vaughan, S.J.

---

The annual dinner of the West London Postgraduate College and past and present members of the West London Hospital, Hammersmith Road, will be held on Wednesday, June 16, at the Trocadero Restaurant, Piccadilly Circus, W., at 7 p.m. for 7.30 p.m. The chair will be taken by P. S. Abraham, Esq., M.D., F.R.C.S.I., dermatologist to the West London Hospital. The price of the dinner ticket is 7s. 6d. (exclusive of wine). All past and present members of the college and hospital are invited to attend, and tickets may be obtained upon applying to the Hon. Secretary, Mr. L. A. Bidwell, F.R.C.S., Dean of the College.

---

The festival dinner in aid of the Metropolitan Hospital, Kingsland Road, N.E., was held at the Whitehall Rooms on May 21 under the presidency of Lord Duncannon. The Chairman reminded his hearers that the institution was not only poor, but also in a poor district. With an average expenditure of £14,000, the assured income from investments barely exceeds £500. Of the 113 beds in the hospital, on an average 105 were in constant occupation during the past year. This year the hospital is faced with the need for extensive repairs to bring it up to modern requirements. These will cost about £8,000, and an isolation ward and a new nurses' home are needed. Upwards of £4,850 was subscribed during the evening.

---

The Incorporated Liverpool School of Tropical Medicine announces that instruction is given in tropical medicine for the diploma in that subject granted by the University of Liverpool, and that the period of instruction is three months. Two courses are held annually from January 6 to April 5 and from September 15 to December 13 respectively. In the summer there is also a short course from June 1 to June 30. At the end of the latter an examination is held and a certificate of attendance is granted to those who pass the examination. This certificate excuses holders from attendance during the first four weeks of the full Lent and autumn courses, one of which is necessary for the diploma. The short course, consisting entirely of practical and clinical laboratory work given at the University laboratory and at the Royal Southern Hospital, is especially designed for medical men at home on leave who have not time to attend the full course for the diploma. The fee for the course is 4 guineas, and applications should be sent to the Dean of the Medical Faculty of the University of Liverpool.

The opening ceremony of the Berlin Tuberculosis Congress took place in that city on Saturday, May 22, when Dr. von Bethmann-Hollweg, Imperial Secretary of State for the Interior, delivered an Inaugural Oration, which dealt principally with the growth and development of institutions for experimental research into the problems connected with the fight against tuberculosis.

---

A Conference arranged by the Invalid Children's Aid Association will be held at Denison House on June 22 and 23. At the four sessions of the conference the chair will be taken by the Duchess of Sutherland, Lord Aberdeen, Dr. Arthur Latham, and Dr. Loch. Programmes, tickets, and other information can be obtained of the Secretary, Invalid Children's Aid Association, 69 Denison House, 296 Vauxhall Bridge Road, Westminster, S.W.

---

The forty-first annual meeting of the Queen's Hospital for Children, Hackney Road, was held at the hospital on May 24, under the chairmanship of Lord William Cecil. The annual report shows that the work of the hospital has increased materially, over 1,700 in-patients and 31,000 out-patients having received treatment. Expenditure, however, exceeded income by £1,300, which, added to an adverse balance in 1907 of £2,100 as well as the £11,000 required for the upkeep of the institution during the present year, throws a heavy responsibility upon the management. King Edward's and the Hospital Sunday and Saturday Funds in recognition of good and economical administration have increased their grants, and the committee appealed with confidence to the public to provide the assistance which is so urgently needed. The committee will be reluctantly compelled to close half the wards unless a strong helping hand is given.

---

Mr. Howard Marsh, M.C., F.R.C.S., Professor of Surgery in the University of Cambridge and the Master of Downing College, presided at the Annual Dinner of the Medical Graduates College and Polyclinic held at the Trocadero Restaurant last week, and was supported among others by Sir W. Church, Sir D. MacAlister, Sir J. Hutchinson, Sir A. H. Keogh, Inspector-General J. Porter, Mr. Bland Sutton, the Master of the Society of Apothecaries, Dr. Fletcher Little, Dr. Theodore Williams, Dr. A. Newsholme, and Dr. T. N. Kelynack. The Chairman said that the Polyclinic had to do pioneer work in providing information for those who had not the time to obtain it for themselves. Polyclinics by the extension through their efforts of improved methods and recent knowledge to medical practitioners in private or public practice are really charitable institutions conducted on the soundest possible lines. Sir Jonathan Hutchinson, F.R.C.S., in support of the Polyclinic movement, pointed out that education does not stop at school or college, but is a lifelong process, which in a sense only begins when the diploma is taken. Dr. C. O. Hawthorne said that the administrator of a polyclinic must display an even greater variety of capacity than was needed in the administrator of a lunatic asylum. He then presented to Captain A. E. Hayward Pinch, F.R.C.S., late Medical Superintendent of the College and now Director of the Radium Institute, a cheque for a hundred guineas and an illustrated address, signed by many leading physicians and surgeons and friends, testifying to the zeal, ability, and judgment with which Captain Pinch during his ten years of office served the interests of the College.

Dr. William Engelmann, Professor of Physiology in the University of Berlin and distinguished for his researches into the neuro-muscular mechanism of the heart, died on May 20, at Berlin, at the age of sixty-five. Professor Engelmann was professor at Utrecht before his removal to Berlin.

The National Hospital for the Paralysed and Epileptic (Albany Memorial), Queen Square, Bloomsbury, W.C., which was founded in 1859, and therefore celebrates its jubilee this year, will hold a festival dinner, with the Right Hon. the Lord Mayor in the chair, on Thursday, June 10, at the Mansion House, at 7 for 7.30 p.m.

At the annual meeting of the Hospital Officers' Association, held at the Gaiety Hotel on May 20, Mr. H. W. Burleigh, Secretary of the Hospital for Epilepsy and Paralysis, etc., Maida Vale, W., was presented with a silver tea and coffee service by members of the Association upon his resignation as editor of the *Hospital Gazette*, and in recognition of his services as such for the past five years.

### THE GENERAL MEDICAL COUNCIL.

The 89th session of the General Medical Council opened on Tuesday, May 25, at the offices in Oxford Street, with Sir Donald Macalister, K.C.B., M.D., the president, in the chair. Dr. Frederick Taylor, as representative of the University of London, in succession to Dr. Pye-Smith, resigned; and Dr. David Knox, as representative of the Faculty of Physicians and Surgeons of Glasgow, in succession to the late Dr. Lindsay Steven, were introduced, and took their seats on the Council.

The President's Opening Address referred to the representations which had been made by him to the Government, through the Lord President of the Privy Council, on various matters regarding which resolutions were passed in November. In accordance with the Lord President's decision to make preliminary inquiries on the subject of the appointment of a Royal Commission to inquire into the evil effects produced by the unrestricted practice of medicine and surgery by unqualified persons, a circular has been issued to medical officers of health asking for information as to the extent of the practice and its effects on the general health in their districts. The President referred to the Council's resolution of November 28 respecting the General Anæsthetics Bill, 1908, which he has forwarded to the Government departments concerned. The resolution supported the proposed restriction of general anæsthesia to qualified medical practitioners. The Council will be called on to consider whether, apart from the saving to existing dentists of their customary practice, it would be in the public interest to confer in future on dentists without medical qualification express legal authority to administer general anæsthetics for dental operations. Since without skilled attention to the state of the patient's health no general anæsthetic is invariably "safe," and in dental practice the anæsthetist is often the operator also, the proposal contained in the Bills referred to is not without some justification in the public interest. In reply to the Registrar's inquiry as to the degree in which effect had been given by the licensing bodies to the Council's recommendation—that candidates for medical qualifications should be required to produce evidence of having received practical instruction in the administration of anæsthetics, nearly all the licensing bodies state that this requirement has already, or will in future be, enforced. The Council considers itself thus justified in its contention that fresh legislation to this end, which to be effective must be penal in character, is neither expedient nor necessary at the present time. The British Dental Association was also referred to in the Address, and also the Draft Charter of the British Medical Association. Some of the powers asked for by the Association appear at first sight to trench on the statutory functions of the Council, and will therefore need careful consideration.

Dr. Langley Browne moved "That representations be made to the Privy Council that it is expedient to confer on the registered practitioners resident in England and Wales the power of returning an additional member to the General Council." He said that the number of registered medical practitioners in England and Wales was 25,168, in Scotland 3,845, and in Ireland 2,658. England and Wales between them had only three direct representatives, and Scotland and Ireland had one each. Dr. McManus seconded the motion, which was adopted, with the following rider:—"That it be a further representation to the Privy Council that the addition in question be not made until the next general election of direct representatives."

Sir Thomas Myles, a member of the Council, was appointed an assistant examiner in surgery to the Apothecaries' Hall, Dublin, for a period of four years, in lieu of Sir Lambert Ormsby, who retires by rotation, his term having expired.

